# Improved Transformer-Based Dual-Path Network with Amplitude and Complex Domain Feature Fusion for Speech Enhancement

**DOI:** 10.3390/e25020228

**Published:** 2023-01-26

**Authors:** Moujia Ye, Hongjie Wan

**Affiliations:** Information Engineering Department, Beijing University of Chemical Technology, Beijing 100029, China

**Keywords:** speech enhancement, time-frequency analysis, neural network, transformer, complex spectrum, amplitude

## Abstract

Most previous speech enhancement methods only predict amplitude features, but more and more studies have proved that phase information is crucial for speech quality. Recently, there have also been some methods to choose complex features, but complex masks are difficult to estimate. Removing noise while maintaining good speech quality at low signal-to-noise ratios is still a problem. This study proposes a dual-path network structure for speech enhancement that can model complex spectra and amplitudes simultaneously, and introduces an attention-aware feature fusion module to fuse the two features to facilitate overall spectrum recovery. In addition, we improve a transformer-based feature extraction module that can efficiently extract local and global features. The proposed network achieves better performance than the baseline models in experiments on the Voice Bank + DEMAND dataset. We also conducted ablation experiments to verify the effectiveness of the dual-path structure, the improved transformer, and the fusion module, and investigated the effect of the input-mask multiplication strategy on the results.

## 1. Introduction

Speech enhancement is an important task in improving the speech signal-to-noise ratio and speech quality, and has a wide range of applications in improving the performance of speech processing systems, mobile communication, and human–computer interaction systems [1]. According to different principles, speech enhancement methods can be divided into traditional methods based on signal processing and deep-learning methods. Traditional methods are generally classified according to the operation domain and can be divided into the time domain, frequency domain, and time-frequency domain. The representative classical algorithms are subspace-based methods [2], spectral subtraction [3], wavelet packets [4], etc.

The development of machine learning has led to the gradual replacement of many traditional speech enhancement methods by neural network-based approaches. Deep learning-based methods can be studied in three ways: input features, model structure, and target optimization. This paper mainly focuses on the study of the model structure.

The continuous development of network structures such as RNNs [5], CNNs [6], and transformer [7] and their successful application in various fields have brought performance improvements to speech enhancement systems. The advantages and disadvantages of RNNs and CNNs as early proposed networks are becoming more and more obvious in practical work, and how to combine the two to achieve excellent performance has become a hot issue. An end-to-end architecture called CRN [8] incorporates CNN and RNN. The sparsity of the CNN makes the model more efficient in terms of data and computational processing power. The use of bidirectional RNNs helps to model the dynamic association between consecutive frames and improves generalization. In [9], the effect of the size and type of RNN network on the CRN structure is investigated, as well as the convolutional layers and skip connections, and, finally, an efficient CRN structure is proposed that can significantly reduce the loss due to reverberation. In addition, a new model of DPCRN [10] that combines Dual-path RNN [5] and CRN was proposed. This model uses two types of RNNs. One is an intra-chunk RNN for estimating the spectrum for a single period. The other is an inter-chunk RNN used to model the spectrum over time. According to the results of the Deep Noise Suppression Challenge in recent years, some multi-stage methods have also combined multiple structures to improve performance. A neural cascade structure [11] consisting of CRN and UNet [12] was proposed by researchers. It is capable of sequentially estimating the amplitude spectrogram, the time-domain signal, and the complex spectrogram of the enhanced speech, and then training the model with a new three-domain loss function. The above work shows that combining CNN and RNN structures in the model can lead to new enhancement effects. This is also demonstrated in our work.

In addition to CRN, studies on the attention mechanism and transformer model also bring new solutions to improve speech enhancement performance. Currently, models with better enhancement performance, such as CRN and UNet, perform poorly in modeling long sequences and are computationally expensive. The attention mechanism can alleviate these problems. In the paper [13], a complex convolution block attention module, CCBAM, was proposed, which improves the modeling capabilities of complex convolution layers by building more informative features. In addition, a module called Stream Axis Self-Attention (ASA) [14] is used as a new module for speech-intensive prediction, which has played a good role in eliminating echo and reverberation.

Transformer [15] is a purely attention-based network that does not use RNNs or CNNs. It is frequently used in NLP and image processing, and some researchers have found its advantages in speech. A Unet-based Conformer network [16] uses temporal attention and frequency attention to learn dimensional information and achieves good performance in speech enhancement. In addition, a two-stage transformer neural network (TSTNN) [17] possessing an improved transformer was proposed for time-domain speech denoising.

Although some approaches to speech processing in the time domain have achieved good results, more studies [18] have shown that the methods based on the frequency domain can often obtain better speech quality, have strong generalization ability and logical interpretation, and are easier to combine with existing speech processing algorithms. Most of the previous frequency-domain-based methods [19] have only used amplitude as the input feature, ignoring phase. However, various studies have shown that the phase still has a large impact on the improvement of speech quality. For example, the method [20] proposes a phase compensation function to modify the phase spectrum to achieve enhancement, and the article [21] decouples amplitude and phase optimization using a two-stage system. All these studies show that improving the estimation of the phase spectrum yields better-enhanced speech quality. To further address this issue, the researchers proposed the ideal complex ratio mask (CRM) [22] to find the real and imaginary parts of the complex spectrum. Better performance will be obtained if the advantages of the above-mentioned CRN and transformer structure can be fully utilized to estimate the CRM. In addition, considering the fusion of multiple features, multi-modal fusion methods [23] can benefit the task by efficiently extracting high-level nonlinear feature representations. In [24], a method for fusing different features is proposed to fully combine the advantages of the features for complex spectral mapping.

Inspired by the above factors, we propose a dual-path network that incorporates amplitude and complex domain features. The proposed network not only does not discard the phase information, but also facilitates the estimation of complex masks by simultaneously learning the amplitude features. In this work, our contributions are as follows.

First, this paper proposes a dual-path network that can simultaneously extract complex spectral features and amplitude feature masks to obtain better-enhanced speech estimation. Second, in the dual-path structure, an attention-aware feature fusion module is used to help the two branches work together and interact with each other for information, thus making it possible to achieve optimal mask estimation. Third, the improved transformer module processes the data from both directions to learn local and global information. Section 2 describes each part of the proposed dual-path network in detail.

We not only compare the proposed method with other methods in our experiments, but also verify the effectiveness of the dual-path structure, the attention-aware fusion module, and the improved transformer module. The network proposed in this paper is based on a mask-based approach, so the effect of different positions of the input and mask multiplication on the system performance is also investigated in the experiments. The detailed results and analysis are given in Section 4.

In addition, Section 3 gives the dataset, setup, and evaluation metrics of the experiments. Section 5 is the discussion section, which gives several observations obtained in this paper. Conclusions and future research directions are given in Section 6.

## 2. Proposed Dual-Path Speech Enhancement Network

**Figure 1:**
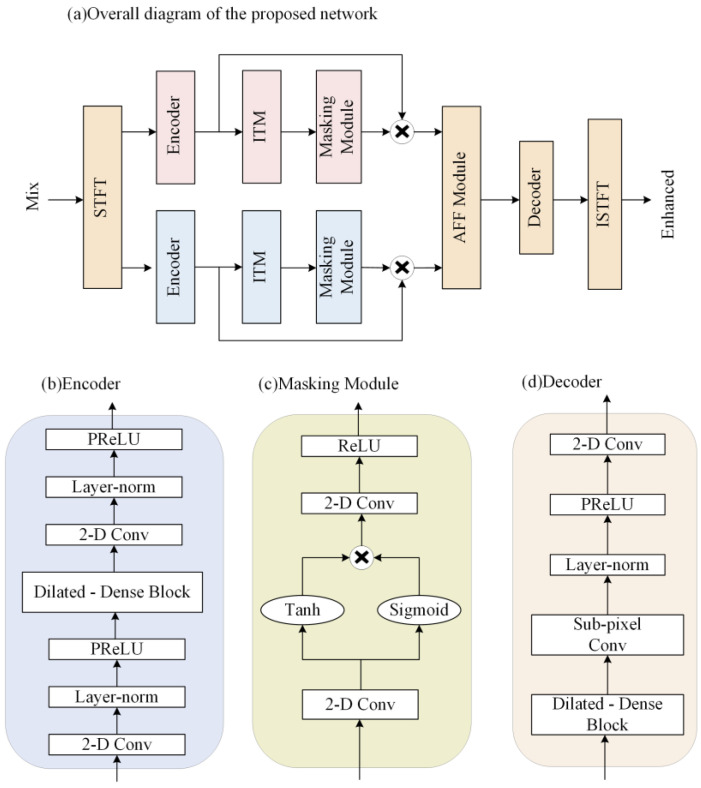
(**a**) Overall structure of the proposed dual-path network. Red rectangles indicate modules that process only complex domain features, blue ones are for amplitude features only, and yellow ones indicate modules that process multiple types of data. (**b**) The internal structure of the encoder. (**c**) The detailed structure of the masking module. (**d**) The detailed structure of the decoder.

In this section, the proposed two-path network, which jointly learns the characteristics of the complex and amplitude domains, is described in detail. As shown in Figure 1a, the proposed network consists of two paths. One path models the features in the complex domain, and the other path models the features in the amplitude domain. These two paths have independent parts and intersecting parts. The independent parts are the respective encoders, the improved transformer modules, and the masking modules, and the intersecting parts are the attention-aware feature fusion module, the decoder, the STFT, and the ISTFT. The network encodes the complex and amplitude separately, and the encoded features are fed to the improved transformer modules for feature extraction, and then the masking modules compute the masks of the two features. Next, the attention-aware feature fusion module fuses the amplitude and complex features after multiplying with the mask to obtain the optimal complex estimate. Then, the features of the same size as the original signal are obtained by the decoder. Finally, the complex features are transformed to obtain the enhanced time-domain signal. A detailed description of each module in Figure 1a will be given in the following subsections.

### 2.1. Encoder

The encoder and decoder structure can efficiently extract the features of the speech signal. The data passing through the encoder has a reduced amount of data per frame, but the number of channels increases, so that more efficient data can be processed using a smaller computational cost, and the data are restored to the original data size by the decoder.

Compared to the complex LSTM, TCNs perform better in modeling long sequences, and has parallelable convolution operations. Thus, training a TCN takes less time. Considering the success of TCNs in speech separation and speaker target extraction, adding it to the enhancement model gives better results. We decided to use a TCN as the main encoder and decoder structure.

The structure of the proposed encoder is improved from the convolutional block in the Conv-TasNet [25]. The detailed network structure of the encoder is shown in Figure 1b. The original convolution block consists of a 2D convolution and a Depthwise convolution (D-conv) [26]. In the modified encoder, we placed the dilated-dense block in the middle of two 2D convolution blocks, and added the PReLU activation function and the normalization operation. The purpose of this is to use 2D convolution to project the input to a higher channel space and trim the data, then use the dilated-dense block to obtain a larger receptive field. The final module obtains smaller features by convolution to save the computational cost of subsequent modules.

### 2.2. Improved Transformer Module

Dual-path RNN [5] and dual-path transformer [27] have successively obtained excellent performance in speech separation tasks. In speech enhancement, feature extraction is very important for the improvement of speech quality. Although the dual-path transformer enables context-aware modeling of speech sequences, its ability to integrate local information is limited. We improve the dual-path transformer and use it as the main feature extraction module of the proposed network. The improved transformer module is able to learn local and global contextual features and does not change the size of the data. It is described in detail in this section.

#### 2.2.1. Improved Transformer

**Figure 2:**
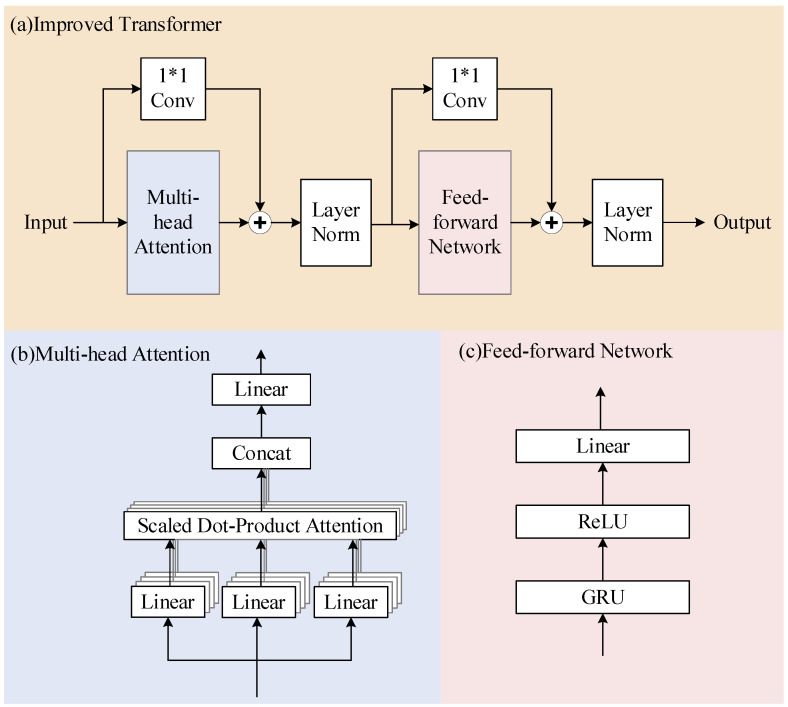
(**a**) The structure of the improved transformer. It has two main parts, namely Multi-head Attention and a feed-forward network. (**b**) The detailed structure of the Multi-head Attention. (**c**) The detailed structure of the feed-forward network.

In previous neural networks, all features received the same attention. However, in the attention mechanism, important features receive more attention, which saves a lot of computational and storage resources. We want the improved transformer to act as an attention module that learns features better rather than doing the whole enhancement work, and therefore use a simplified structure consisting of a Multi-head Attention layer and a feed-forward layer. The specific structure is shown in Figure 2. In deep networks, residual connections can solve the problem of gradient exploding and gradient vanishing in training. In order to reduce the information loss and obtain more local and global information, referring to the results of the paper [9], we add residual connections with 1*1 convolution to the Multi-head Attention layer and feed-forward layer, as shown in Figure 2a.

The mathematical model of the improved transformer is as follows. X is the input data and Y is the output. Letting MultiHeadAttention() be the Multi-head Attention function and FFN() be the feed-forward layer function, then

(1) Head=MultiHeadAttention(X)

(2) Mid=LayerNorm(conv(X)+Head)

(3) Y=LayerNorm(conv(Mid)+FFN(Mid))

Multi-head Attention connects features extracted by different Single-head Attention layers to obtain the final output. This allows Multi-headed Attention to focus on information from different locations that represent features in different subspaces. In the proposed model, we set the number of times to find Single-head Attention, which is also the number of parallel attention layers, to four.

The feed-forward layer of the original transformer is a two-layer, fully connected layer. Such a feed-forward layer is not suitable for learning the location information of speech sequences. Considering the learning ability of RNN in time series, GRU is used as the first layer in the feed-forward layer. The second layer uses the ReLU function, which can significantly alleviate the gradient vanishing problem of the deep network and accelerate the convergence speed of gradient descent. Finally, the linear layer is used as the third layer of the feed-forward network.

#### 2.2.2. The Architecture of Improved Transformer Module

**Figure 3:**
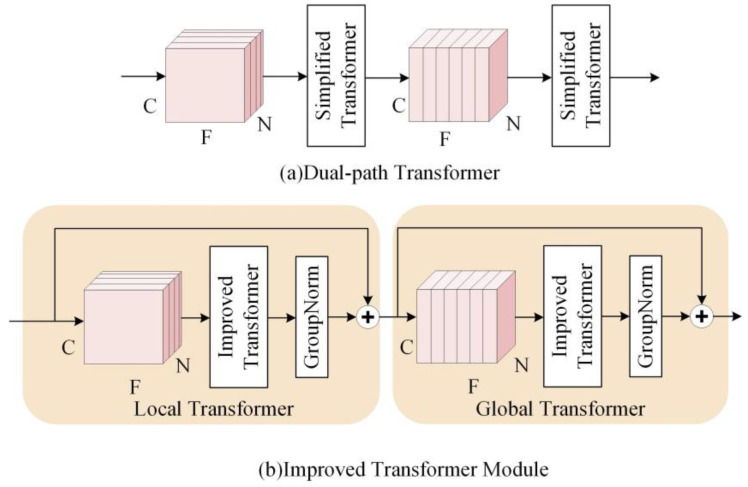
The structure of two types of transformer modules. (**a**) Dual-path transformer. (**b**) Improved transformer module.

Figure 3a shows the two-path transformer proposed in the paper [27], which has an insufficient ability to integrate local information. Therefore, based on the improved transformer, an improved transformer module (ITM) is used in this paper. As shown in Figure 3b, it consists of two identical modules, including the improvement transformer and the group normalization layer. The input data are first sliced to obtain 3D data (C, N, F). N denotes the number of frames, F is the amount of data per frame, and C is the number of channels. Then, the first transformer, called the local transformer, processes the data in the last dimension of the tensor and learns the local information in parallel. Then, the second transformer, called the global transformer, processes the data in the second dimension of the tensor, fusing the local information and learning the connections between the data blocks.

### 2.3. Attention-Aware Feature Fusion Module

**Figure 4:**
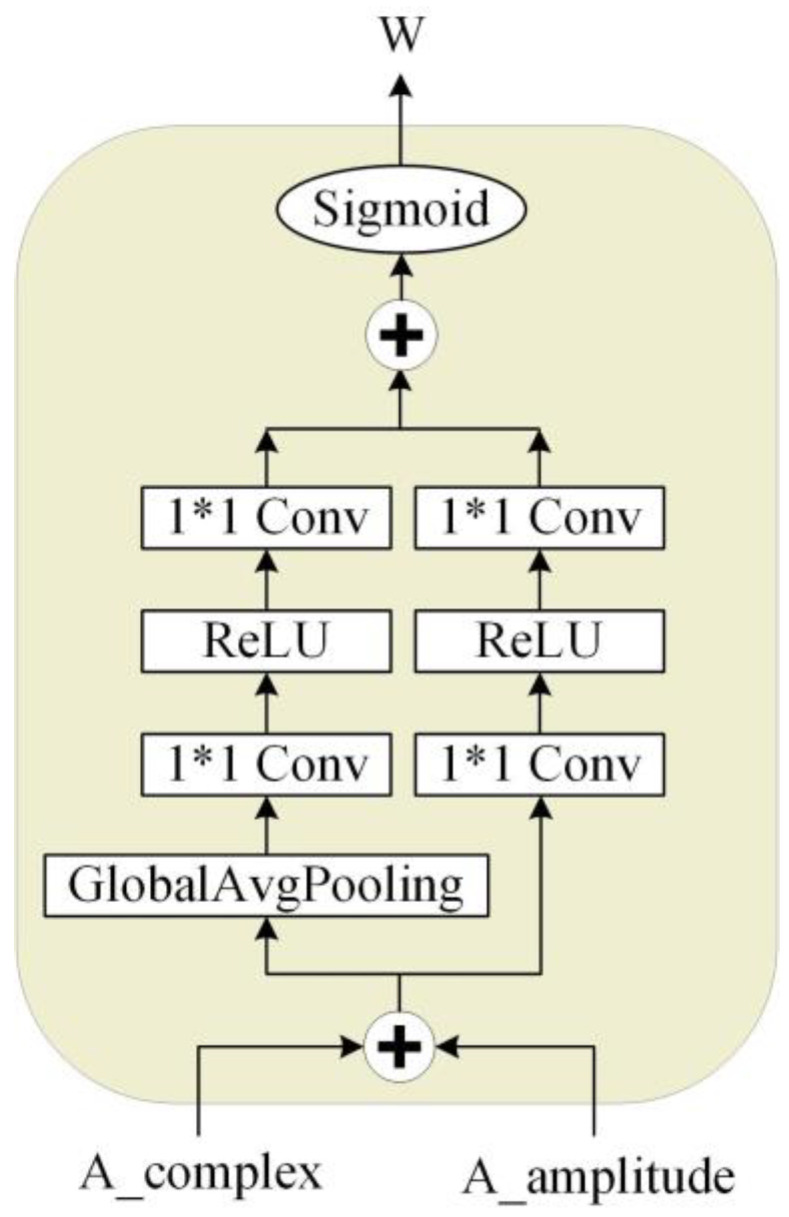
Attention networks used in the attention-aware feature fusion module.

The attention-aware feature fusion (AFF) module is proposed in this paper to fuse the amplitude and complex features learned from the dual-path structure to obtain the best spectral estimate. From Equations (4) and (6), we obtain the complex and amplitude features corrected by the mask. The amplitude and phase information found by the complex number branch can be obtained according to Equation (5). Equation (7) shows how to fuse the amplitude features of the complex branch with the features of the amplitude branch. The parameter W is obtained from the attention-aware network, the detailed structure of which is shown in Figure 4. Finally, the final complex spectrum is calculated using the fused amplitude combination according to Equation (8).

(4) $${\overset{⌢}{C}}_{R}^{}+i{\overset{⌢}{C}}_{I}^{}=(C_{R}^{}+iC_{I}^{})*(H_{R}^{}+iH_{I}^{})$$

(5) $$A_{C}=\sqrt{{\overset{⌢}{C}}_{R}^{}{}^{2}+{\overset{⌢}{C}}_{I}^{}{}^{2}},P_{C}=\arctan({\overset{⌢}{C}}_{I}^{}/{\overset{⌢}{C}}_{R}^{})$$

(6) $${\overset{⌢}{A}}_{}^{}=A_{A}^{}*H_{A}$$

(7) $$A_{AFF}^{}=W*A_{C}^{}+(1-W)*\overset{⌢}{A}$$

(8) $$Y_{R}^{}=A_{AFF}^{}\cdot \cos P_{C},Y_{I}^{}=A_{AFF}^{}\cdot \sin P_{C}$$

where $A_{A}$, $C_{R}^{}$, and $C_{I}^{}$ denote the amplitude, complex real, and imaginary parts after encoding. $H_{A}^{}$, $H_{R}^{}$, and $H_{I}^{}$ denote their masks. $\overset{⌢}{A}$, ${\overset{⌢}{C}}_{R}^{}$, and ${\overset{⌢}{C}}_{I}^{}$ are after mask correction; $A_{AFF}^{}$ is the amplitude feature after fusion obtained by attention weights, and $Y_{R}^{}$ and $Y_{I}^{}$ are the complex feature after fusion.

The attention network used by the AFF module is similar to the multi-modal fusion module proposed in the paper [28]. Ours is characterized by the use of a two-branch network with global extractors and local extractors. The local extractor consists of two layers of point-wise convolution and a ReLU activation function. The global extractor adds a global average pooling layer to the local extractor. This attention network can combine local and global information to give optimal weights. 

### 2.4. Masking Module

First, the contextual relevance of the speech signal is considered to have an important impact on speech quality. It can effectively improve speech quality if longer speech sequences can be modeled. Secondly, the temporal convolution (TCN) module has been shown to outperform RNNs in modeling long sequences.

Considering the above two factors, we improved the TCN-based S-TCM [29] as a masking module. The masking module generates masks after learning the features extracted by the ITM. The structure is shown in Figure 1c. Convolution and activation functions are used before and after the double branch to obtain an accurate estimation of the mask. The module uses different activation functions in the double branch. The Tanh function is the activation function of the main branch to speed up the convergence and avoid the gradient exploding problem. Sigmoid is used as the activation function for the gated branch, adjusting the output value to (0, 1), which allows a better flow of information in the gradient propagation.

### 2.5. Decoder

The structure of the decoder is shown in Figure 1d. It consists of a dilated-dense block, subpixel convolution [30], normalization layer, PReLU activation function, and 2D convolution layer. The decoder uses the dilated-dense block and subpixel convolution to obtain the up-sampled data. Normalization and activation functions make the data more normalized. Then, 2D convolution is used to change the multichannel data into a single-channel speech frame. The function of the decoder is to reconstruct the processed features to obtain the same size data as the input. After these data are summed by inverse STFT and overlap-add, we can obtain enhanced speech.

### 2.6. Loss Function

The loss function of the proposed dual-path network combines the loss functions in the time domain and time-frequency domain. The loss function ensures that the error between the estimated results and the clean complex domain features is minimized, obtaining better speech intelligibility and perceptual quality, as defined below:

(9) $$loss_{F}=\frac{1}{TF}\sum_{t=0}^{T-1}\sum_{f=0}^{F-1}\left[\left(\left|X_{r}(t,f)\right|+\left|X_{i}(t,f)\right|\right)-\left(\left|{\overset{⌢}{X}}_{r}(t,f)\right|+\left|{\overset{⌢}{X}}_{i}(t,f)\right|\right)\right]$$

where $X_{r}(t,f)$, $X_{i}(t,f)$, ${\overset{⌢}{X}}_{r}(t,f)$, ${\overset{⌢}{X}}_{i}(t,f)$ denote the real and imaginary parts of the spectrum of the clean waveform, and the real and imaginary parts of the spectrum of the enhanced waveform, at the time frame *t* and frequency index *f*, respectively. *T* and *F* are the number of time frames and frequency bins. The enhanced speech frames are converted to waveforms using overlap-add, and the loss is calculated in the time domain using the mean square error between the enhanced and clean speech. The time-domain loss function [31] is defined as follows:

(10) $$loss_{T}=\frac{1}{N}{\sum_{i=0}^{N-1}\left(x_{i}-{\overset{⌢}{x}}_{i}\right)}^{2}$$

where $x_{i}$ and ${\overset{⌢}{x}}_{i}$ denote clean and noise signals of the time index i. The N is the number of samples. The loss function used in this paper is obtained according to the following equation:

(11) $$loss=\alpha *loss_{F}+\left(1-\alpha \right)loss_{T}$$

where α is an adjustable parameter and is set to 0.2 in this experiment.

## 3. Experimental Setup

### 3.1. Datasets

The proposed dual-path network was trained and tested on the Voice Bank + DEMAND dataset [32]. The clean speech of this dataset is obtained from the Voice Bank corpus, and 5000 utterances from 28 speakers are used for the training set; the noisy speech is generated from clean speech and 10 noises from the DEMAND dataset in SNR levels [0, 5, 10, 15]. The clean speech of the test set consists of 824 voices from two speakers, and the noisy speech is obtained by mixing clean speech and five unseen noises at SNR levels of [2.5, 7.5, 12.5, 17.5]. In order to perform a valid and reasonable evaluation of the proposed model, noisy test and training data with different original clean speech, SNRs, and noise are used in this experiment.

### 3.2. Training Setup

In this experiment, all utterances are resampled to 16 kHz. If the speech is larger than 4 seconds, a random 4-second segment is selected, and if it is less than 4 seconds, the speech is repeated for filling. Then, the speech data are framed, with each frame having a size of 512 data values with an overlap of 256 data values.

The model is trained with a maximum epoch of 40 and uses the Adam optimizer. The learning rate setting is very important. Using too large a learning rate for the model may not lead to convergence, and too small may take too much time. If a piecewise decreasing learning rate is used, the learning ability of the model may fluctuate drastically when using mini-batch data fed into the network. This is not conducive to the deep stability of the model. Considering the above analysis, we use a dynamic decay strategy [7] with two stages. In the first stage, the learning rate increases linearly from a very small learning rate to the base learning rate. In the second stage, it decays by 0.98 every two epochs starting from the base learning.

### 3.3. Evaluation Metrics

We evaluated the proposed dual-path network on two aspects, the denoising effect and speech quality. The objective metric used for the denoising effect is the signal-to-noise ratio (SNR). The metrics used to assess speech quality are the perceptual evaluation of speech quality (PESQ) [33] and the subjective mean opinion score (MOS) [34]. The MOS includes CSIG for signal distortion, CBAK for noise distortion assessment, and COVL for overall quality assessment. All MOSs range from 1 to 5.

## 4. Results and Analysis

### 4.1. Comparison with Other Methods

The baseline model is a mask-based approach using a transformer module similar to the proposed method. Compared to this model, the proposed model has a dual-path structure that learns both features simultaneously and a fusion module that uses an attention mechanism to fuse the two features, and adds 1*1 convolution to the improved transformer to help integrate information and add nonlinearity. For the baseline models, we used the TSTNN model with 2 TSTMs and the one with 4 TSTMs. For the proposed model, we used one ITM and stacked two ITMs, respectively. All models were trained and tested under the same conditions.

#### 4.1.1. Objective Metrics Comparison

**Figure 5:**
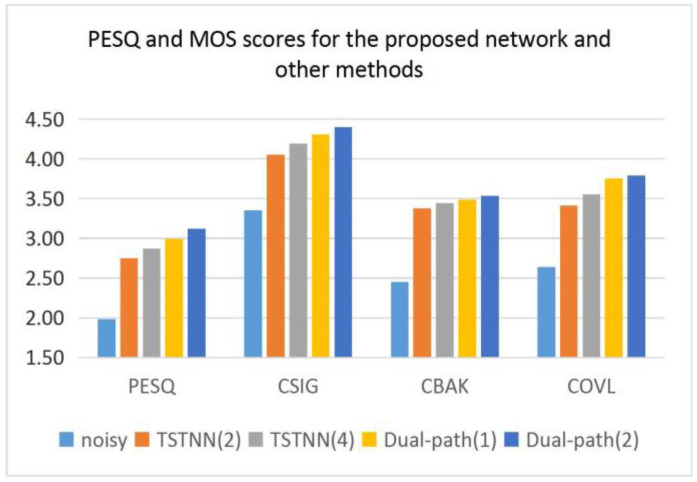
PESQ and MOS scores for the proposed network and other methods. The data in the figure are the average values obtained from all the measured voices.

It can be seen from Figure 5 that the proposed network has better test results than the other models in terms of both PESQ and MOS scores. This is a good indication that it can obtain better spectral estimation, which leads to better speech quality. This may be the result of a strategy for the proposed network to learn both characteristics at the same time and integrate them.

According to Figure 5, Table 1 and Table 2, the number of parameters of the TSTNN with two TSTMs is similar to that of the proposed network, but it scores lower than the proposed network in all metrics. The TSTNN with four TSTMs has more parameters than that of the proposed network, but the proposed network scores higher on most of the metrics. This clearly shows that the proposed network has better performance and a smaller model size.

In addition, the above results can show that another advantage of the proposed network is that we can balance the performance and computational resources well by changing the number of ITMs. From the results, the model with two ITMs has a higher score than the model with only one. More ITMs mean better performance. If computational cost and parameter storage space are limited, we can use only one ITM. Conversely, if better performance is needed, more ITMs can be stacked.

#### 4.1.2. Enhanced Spectrogram Comparison

**Figure 6:**
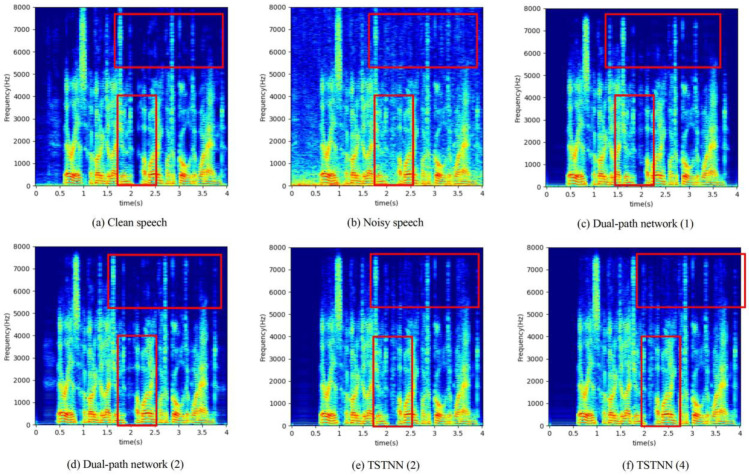
Spectrograms of clean speech, noisy speech, and enhanced speech obtained by proposed networks and other methods.

To explain more intuitively the effectiveness of the proposed network, Figure 6 shows the spectrograms of clean speech, noisy speech, and enhanced speech of the proposed networks and comparison methods, respectively. The noisy speech example is randomly selected from the test set and is obtained by mixing clean speech and cafe noise at an SNR of 7.5 dB. The red markers highlight the obvious differences between each speech spectrogram. For the part above 5 kHz, it is clear that the proposed network removes more noise. The proposed network with two ITMs is the closest to the clean speech spectrogram and has the least degradation. For the 0–4 kHz part, it is also found that the proposed network has better noise removal and less speech distortion.

### 4.2. Ablation Experiments

The results in the previous subsection show that the proposed dual-path network improves the speech quality and signal-to-noise ratio compared to other methods. To further validate the validity of the submodules of the proposed model, we conducted a three-part ablation experiment. First, we verify the superiority of the dual-path network over the single-path network. Then, the necessity of ITMs and the validity of the AFF module are verified.

#### 4.2.1. The Superiority of Dual-Path Structure

**Figure 7:**
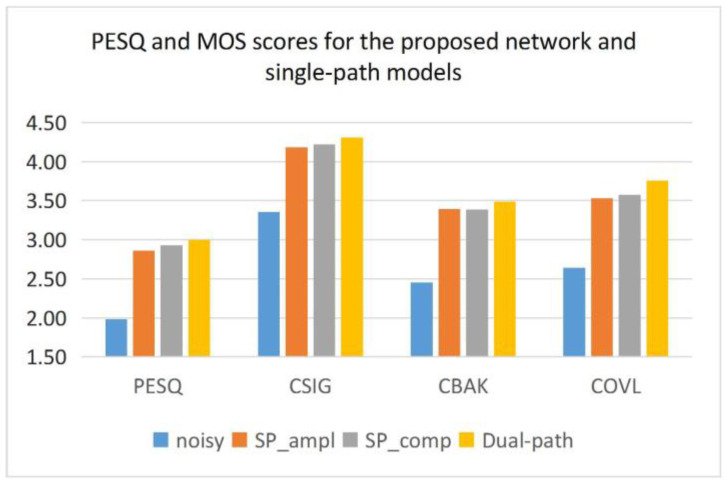
PESQ and MOS scores for the proposed network and single-path models.

The proposed method uses a dual-path structure to learn two features separately and fuse them to promote each other’s learning for better results. In order to verify whether the dual path has advantages over the single path, this paper uses the single path to model the complex and amplitude features separately and compares the results of the three models. In Figure 7 and Table 3, ‘SP_comp’ denotes the method of modeling complex features using a single path, and ‘SP_ampl’ denotes the method of modeling amplitude features using a single path.

As can be seen from Figure 7 and Table 3, the dual-path model outperforms the single-path complex model and the single-path amplitude model for all metrics in terms of the PESQ and MOS. The worst of them is the single-path amplitude model. This is a good indication that the proposed dual-path network can effectively improve speech quality, and the mapping using complex features is better than the mapping using only amplitude features. This also shows the importance of phase information in terms of speech quality. In terms of the SNR, the two-path network performs optimally at 2.5–12.5 dB, and only the single-path network using amplitude characteristics at 20 dB has the best metrics. However, this advantage is not significant and loses speech quality as a cost.

#### 4.2.2. The Necessity of Improved Transformer Module

In this paper, we improve the transformer to obtain the ITM, which can extract local and global information. To verify the effectiveness of the ITM module, we designed a comparison. The model for comparison removes the ITM module and increases the number of layers of encoder and decoder to two layers. The purpose of this is to use the convolutional layer in the encoder instead of the ITM for feature learning to determine whether it is necessary for the ITM to exist in the network.

**Figure 8:**
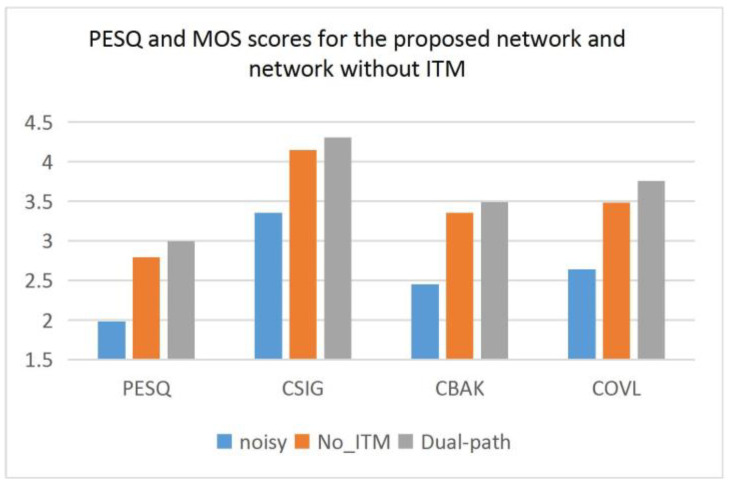
PESQ and MOS scores for the proposed network and network without ITM.

As can be seen from Table 4 and Figure 8, the proposed model performs better in all metrics compared to the model with the ITM replaced by convolutional layers, especially in the PESQ and MOS metrics. This fully demonstrates that the ITM module can effectively extract features to help improve speech quality.

#### 4.2.3. The Effectiveness of Attention-aware Feature Fusion Module

In this section, to fully verify the validity of AFF, we set the value of the weight W in Equation (7) to 0.5, which means that the amplitudes of the two features are equally divided. The weight W is obtained in the proposed model by AFF learning the two features.

**Figure 9:**
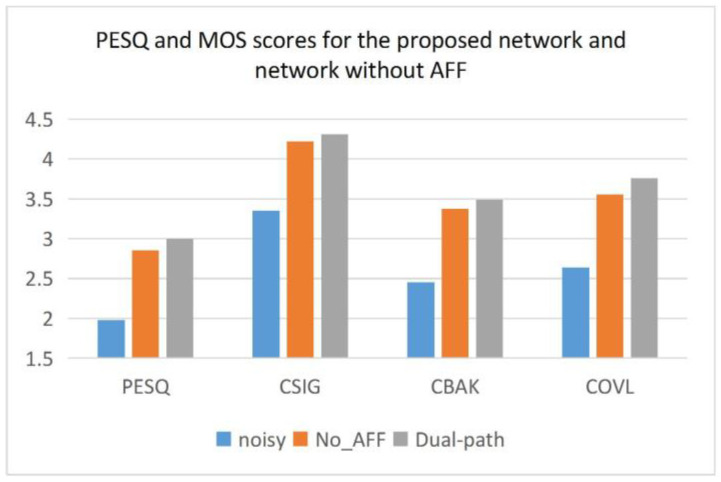
PESQ and MOS scores for the proposed network and network without AFF.

From Table 5 and Figure 9, it can be seen that the model without AFF can achieve some enhancement effect on noisy speech, but its test results are worse than the proposed model in all evaluation metrics, which fully demonstrates that the AFF module can enhance the enhancement performance of the system.

### 4.3. Different Placements of Multiplication Modules on the Effectiveness of the Mask-based Approach

The above results fully demonstrate the superiority of the proposed model among all models. Considering that the proposed model uses a mask-based approach, we conducted experiments to further verify the effect of different placements of the multiplication operation on the mask-based approach.

**Figure 10:**
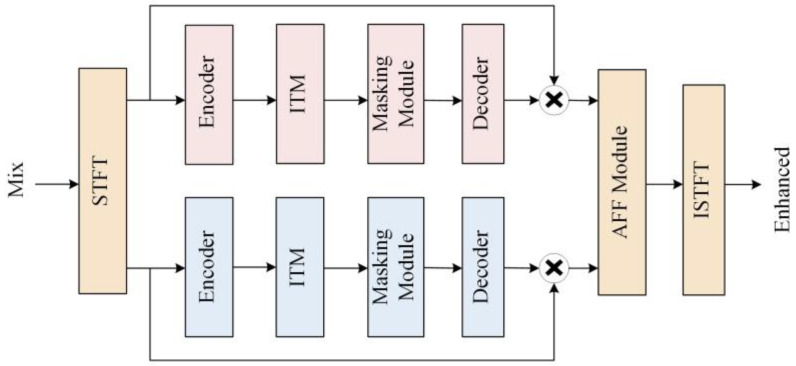
The network structure using the decode-then-multiply strategy.

**Figure 11:**
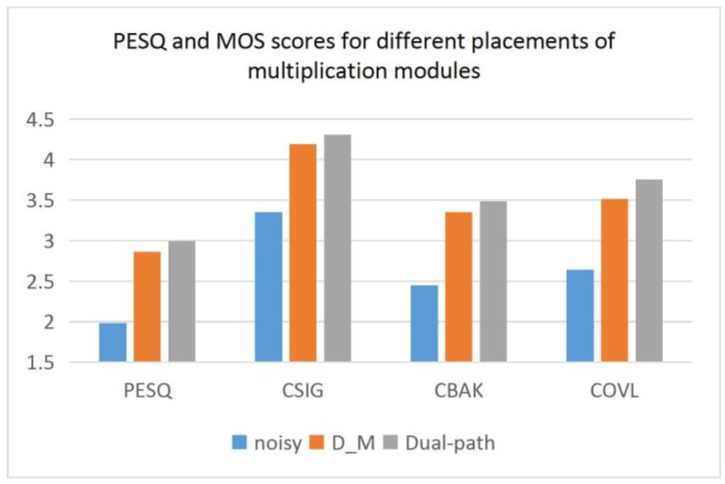
PESQ and MOS scores for different placements of multiplication modules.

The network proposed in this paper uses a dual-path architecture to learn two features simultaneously and has an encoder and decoder. This poses a problem in that the different placement of the multiplication module changes the number of decoders. The multiplication module of the proposed method is before the decoder, and only one decoder is needed to decode the complex features. If the decoder is before the multiplication module, two decoders are needed to decode the complex and amplitude features separately, and then fuse and multiply them. Firstly, it is a change in storage, and secondly, it changes how it affects the performance. These are the questions worth thinking about. This design uses the model of the decoder before the multiplication module as a comparison, and the specific structure is shown in Figure 10. In Figure 11 and Table 6, ‘D_M’ denotes the network of the decoder module in front of the multiplication module.

Figure 11 shows that the method of multiplying before decoding has better performance in terms of both the PESQ and MOS, which illustrates the advantage of this method in terms of speech quality. The data in Table 6 show that the decode-then-multiply approach has a better denoising effect at a high SNR, but this advantage is not obvious. We consider the computational cost and finally choose to place the fusion multiplication module before the decoder.

## 5. Discussion

In this paper, an improved transformer network is proposed, and the superiority of the proposed dual-path model in terms of denoising effect and speech quality is verified through a series of experiments on the Voice Bank + DEMAND dataset. After analyzing the experimental results, some observations are given in the following.

Compared to the single-path model, our approach offers consistent advantages over the single-path model for most of the metrics tested. The reasons considered are as follows. Compared to the single-path model that learns only the amplitude and the single-path model that learns only the complex spectral features, the proposed dual-path model not only learns both features, but also enables effective information interaction between the two, which gives the model a better learning capability.

The proposed network has a better performance compared to the model using convolutional layers instead of an ITM. This well illustrates that using a transformer between the encoder and decoder to model the features is a better choice than adding more convolutional layers. One reason for our consideration is that CNNs focus only on the interconnections between two-dimensional local data. Our improved transformer module can take advantage of the correlation between the whole and the local, which is beneficial for speech spectrum feature extraction. In addition, the use of AFF for feature fusion improves the system performance more than using fixed weights, which may be due to the fact that AFF has an attention-aware network that learns the potential relationship between two features and gives weights adaptively.

For the mask-based DNN speech enhancement method, experiments are conducted in this paper to discuss the effect of the position of the input multiplied with the mask on the model performance. With the results in Section 4.3, we find that decoding before multiplying does not lead to better performance of the proposed model and imposes a greater computational burden.

In summary, our proposed dual-path network chooses the optimal strategy of multiplying masks with inputs. It outperforms the single-path baseline models with a transformer in most metrics and has fewer parameters.

## 6. Conclusions

This paper proposes an improved transformer-based dual-path speech enhancement network with amplitude and complex feature fusion. The network has two paths, modeling both complex spectrum and amplitude, and uses a fusion module for information interaction and improved transformer modules to fully extract features. We used the Voice Bank + DEMAND dataset to train and test the proposed network. The results show that the proposed network has better speech quality performance and fewer trainable parameters compared to the baseline models. In addition, ablation experiments validate the necessity of two-path networks, improved transformers, and attention-aware feature fusion, and some observations about mask-based enhancement methods are given.

In the future, we will modify the modeling module to accommodate the characteristics of different features, instead of using the same structure. In this way, we expect to obtain more accurate spectral information and improve speech quality. In addition, we will also study the performance of our method in complex environments where noise, reverberation, and speaker interference are all present.

## Figures and Tables

**Table 1:** The performance of the proposed network and comparison models.

	PESQ				CSIG				CBAK				COVL				SNR			
SNR	2.50	7.50	12.50	17.50	2.50	7.50	12.50	17.50	2.50	7.50	12.50	17.50	2.50	7.50	12.50	17.50	2.50	7.50	12.50	17.50
Noisy	1.43	1.77	2.11	2.61	2.62	3.15	3.59	4.05	1.79	2.22	2.63	3.17	1.96	2.42	2.84	3.34	1.75	6.28	10.97	15.94
TSTNN(2)	2.20	2.65	2.90	3.26	3.54	3.97	4.20	4.50	2.95	3.30	3.50	3.76	2.86	3.32	3.56	3.91	15.54	17.98	19.39	20.28
TSTNN(4)	2.33	2.77	3.02	3.38	3.68	4.11	4.35	4.64	3.03	3.37	3.57	3.82	3.00	3.45	3.70	4.05	15.80	18.16	19.35	20.66
Dual-path(1)	2.45	2.91	3.14	3.49	3.83	4.24	4.45	4.72	3.08	3.42	3.61	3.84	3.14	3.59	4.15	4.15	15.73	18.13	19.41	20.65
Dual-path(2)	**2.56**	**3.03**	**3.28**	**3.61**	**3.92**	**4.33**	**4.56**	**4.80**	**3.16**	**3.47**	**3.65**	**3.87**	**3.24**	**3.70**	**3.95**	**4.26**	**15.92**	**18.24**	**19.51**	**20.76**

**Table 2:** Number of trainable parameters for the proposed network and comparison models.

	TSTNN(2)	TSTNN(4)	Dual-Path(1)	Dual-Path(2)
Param(million)	0.7401	0.9248	0.6602	0.7525

**Table 3:** The performance of the proposed network and single-path models.

	PESQ				CSIG				CBAK				COVL				SNR			
SNR	2.50	7.50	12.50	17.50	2.50	7.50	12.50	17.50	2.50	7.50	12.50	17.50	2.50	7.50	12.50	17.50	2.50	7.50	12.50	17.50
Noisy	1.43	1.77	2.11	2.61	2.62	3.15	3.59	4.05	1.79	2.22	2.63	3.17	1.96	2.42	2.84	3.34	1.75	6.28	10.97	15.94
SP_ampl	2.32	2.75	3.03	3.35	3.66	4.08	4.36	4.62	2.96	3.30	3.53	3.79	2.98	3.42	3.71	4.02	15.21	17.69	19.16	**20.82**
SP_comp	2.38	2.83	3.11	3.41	3.70	4.12	4.43	4.63	2.96	3.36	3.56	3.66	2.99	3.43	3.79	4.09	15.44	17.90	19.00	19.96
Dual-path	**2.45**	**2.91**	**3.14**	**3.49**	**3.83**	**4.24**	**4.45**	**4.72**	**3.08**	**3.42**	**3.61**	**3.84**	**3.14**	**3.59**	**4.15**	**4.15**	**15.73**	**18.13**	**19.41**	20.65

**Table 4:** The performance of the proposed network and network without ITM.

	PESQ				CSIG				CBAK				COVL				SNR			
SNR	2.50	7.50	12.50	17.50	2.50	7.50	12.50	17.50	2.50	7.50	12.50	17.50	2.50	7.50	12.50	17.50	2.50	7.50	12.50	17.50
noisy	1.43	1.77	2.11	2.61	2.62	3.15	3.59	4.05	1.79	2.22	2.63	3.17	1.96	2.42	2.84	3.34	1.75	6.28	10.97	15.94
No_ITM	2.23	2.70	2.95	3.30	3.60	4.07	4.30	4.60	2.93	3.29	3.48	3.71	2.91	3.40	3.64	3.98	15.22	17.69	18.98	19.96
Dual-path	**2.45**	**2.91**	**3.14**	**3.49**	**3.83**	**4.24**	**4.45**	**4.72**	**3.08**	**3.42**	**3.61**	**3.84**	**3.14**	**3.59**	**4.15**	**4.15**	**15.73**	**18.13**	**19.41**	**20.65**

**Table 5:** The performance of the proposed network and network without AFF.

	PESQ				CSIG				CBAK				COVL				SNR			
SNR	2.50	7.50	12.50	17.50	2.50	7.50	12.50	17.50	2.50	7.50	12.50	17.50	2.50	7.50	12.50	17.50	2.50	7.50	12.50	17.50
Noisy	1.43	1.77	2.11	2.61	2.62	3.15	3.59	4.05	1.79	2.22	2.63	3.17	1.96	2.42	2.84	3.34	1.75	6.28	10.97	15.94
No_AFF	2.33	2.77	3.01	3.32	3.73	4.15	4.38	4.64	2.99	3.32	3.49	3.70	3.03	3.47	3.71	4.01	15.30	17.51	18.50	19.41
Dual-path	**2.45**	**2.91**	**3.14**	**3.49**	**3.83**	**4.24**	**4.45**	**4.72**	**3.08**	**3.42**	**3.61**	**3.84**	**3.14**	**3.59**	**4.15**	**4.15**	**15.73**	**18.13**	**19.41**	**20.65**

**Table 6:** The performance of different placements of multiplication modules.

	PESQ				CSIG				CBAK				COVL				SNR			
SNR	2.50	7.50	12.50	17.50	2.50	7.50	12.50	17.50	2.50	7.50	12.50	17.50	2.50	7.50	12.50	17.50	2.50	7.50	12.50	17.50
Noisy	1.43	1.77	2.11	2.61	2.62	3.15	3.59	4.05	1.79	2.22	2.63	3.17	1.96	2.42	2.84	3.34	1.75	6.28	10.97	15.94
D_M	2.34	2.77	3.00	3.37	3.53	3.99	4.26	4.57	2.95	3.31	3.51	3.83	2.91	3.38	3.63	4.01	15.63	18.10	19.33	**21.35**
Dual-path	**2.45**	**2.91**	**3.14**	**3.49**	**3.83**	**4.24**	**4.45**	**4.72**	**3.08**	**3.42**	**3.61**	**3.84**	**3.14**	**3.59**	**4.15**	**4.15**	**15.73**	**18.13**	**19.41**	20.65

## Data Availability

The data supporting the conclusions of this paper are available at the CSTR VCTK Corpus (https://datashare.ed.ac.uk/handle/10283/2651 (accessed on 29 May 2022)) and the Demand database (http://parole.loria.fr/DEMAND/ (accessed on 29 May 2022)).

## References

[1] Taherian H., Wang Z.Q., Chang J., Wang D. (2020). Robust speaker recognition based on single-channel and multi-channel speech enhancement. IEEE/ACM Trans. Audio Speech Lang. Process..

[2] Hu Y., Loizou P.C. (2003). A generalized subspace approach for enhancing speech corrupted by colored noise. IEEE/ACM Trans. Audio Speech Lang. Process..

[3] Boll S. (1979). Suppression of acoustic noise in speech using spectral subtraction. IEEE/ACM Trans. Audio Speech Lang. Process..

[4] Donoho D.L. (1995). De-noising by soft-thresholding. IEEE Trans. Inf. Theory.

[5] Luo Y., Chen Z., Yoshioka T. Dual-Path RNN: Efficient Long Sequence Modeling for Time-Domain Single-Channel Speech Separation. Proceedings of the ICASSP 2020—2020 IEEE International Conference on Acoustics, Speech and Signal Processing (ICASSP).

[6] Fu S.-W., Tsao Y., Lu X. SNR-aware convolutional neural network modeling for speech enhancement. Proceedings of the INTERSPEECH 2016.

[7] Vaswani A., Shazeer N., Parmar N., Uszkoreit J., Jones L., Gomez A.N., Kaiser L., Polosukhin I. (2017). Attention is all you need. arXiv.

[8] Zhao H., Zarar S., Tashev I., Lee C.-H. Convolutional-Recurrent Neural Networks for Speech Enhancement. Proceedings of the 2018 IEEE International Conference on Acoustics, Speech and Signal Processing (ICASSP).

[9] Braun S., Gamper H., Reddy C.K.A., Tashev I. Towards Efficient Models for Real-Time Deep Noise Suppression. Proceedings of the ICASSP 2021—2021 IEEE International Conference on Acoustics, Speech and Signal Processing (ICASSP).

[10] Le X., Chen H., Chen K., Lu J. (2021). DPCRN: Dual-Path Convolution Recurrent Network for Single Channel Speech Enhancement. arXiv.

[11] Wang H., Wang D. (2022). Neural cascade architecture with triple-domain loss for speech enhancement. IEEE/ACM Trans. Audio Speech Lang. Process..

[12] Weng W., Zhu X. (2021). INet: Convolutional Networks for Biomedical Image Segmentation. IEEE Access.

[13] Zhao S., Nguyen T.H., Ma B. Monaural Speech Enhancement with Complex Convolutional Block Attention Module and Joint Time Frequency Losses. Proceedings of the ICASSP 2021—2021 IEEE International Conference on Acoustics, Speech and Signal Processing (ICASSP).

[14] Zhang G., Wang C., Yu L., Wei J. Multi-Scale Temporal Frequency Convolutional Network with Axial Attention for Multi-Channel Speech Enhancement. Proceedings of the ICASSP 2022—2022 IEEE International Conference on Acoustics, Speech and Signal Processing (ICASSP).

[15] Subakan C., Ravanelli M., Cornell S., Bronzi M., Zhong J. Attention Is All You Need in Speech Separation. Proceedings of the ICASSP 2021—2021 IEEE International Conference on Acoustics, Speech and Signal Processing (ICASSP).

[16] Fu Y., Liu Y., Li J., Luo D., Lv S., Jv Y., Xie L. Uformer: A Unet Based Dilated Complex & Real Dual-Path Conformer Network for Simultaneous Speech Enhancement and Dereverberation. Proceedings of the ICASSP 2022—2022 IEEE International Conference on Acoustics, Speech and Signal Processing (ICASSP).

[17] Wang K., He B., Zhu W.P. TSTNN: Two-Stage Transformer Based Neural Network for Speech Enhancement in the Time Domain. Proceedings of the ICASSP 2021—2021 IEEE International Conference on Acoustics, Speech and Signal Processing (ICASSP).

[18] Xu Y., Du J., Dai L.-R., Lee C.-H. (2015). A regression approach to speech enhancement based on deep neural networks. IEEE/ACM Trans. Audio Speech Lang. Process..

[19] Erdogan H., Hershey J.R., Watanabe S., Roux J.L. Phase-sensitive and recognition-boosted speech separation using deep recurrent neural networks. Proceedings of the 2015 IEEE International Conference on Acoustics, Speech and Signal Processing (ICASSP).

[20] Lavanya T., Nagarajan T., Vijayalakshmi P. (2020). Multi-Level Single-Channel Speech Enhancement Using a Unified Framework for Estimating Magnitude and Phase Spectra. IEEE/ACM Trans. Audio Speech Lang. Process..

[21] Li A., Liu W., Luo X., Zheng C., Li X. ICASSP 2021 Deep Noise Suppression Challenge: Decoupling Magnitude and Phase Optimization with a Two-Stage Deep Network. Proceedings of the ICASSP 2021—2021 IEEE International Conference on Acoustics, Speech and Signal Processing (ICASSP).

[22] Williamson D.S., Wang Y., Wang D. (2016). Complex ratio masking for monaural speech separation. IEEE/ACM Trans. Audio Speech Lang. Process..

[23] Wang Y. (2021). Survey on Deep Multi-modal Data Analytics: Collaboration, Rivalry, and Fusion. ACM Trans. Multimed. Comput. Commun. Appl. (TOMM).

[24] Wang H., Zhang X., Wang D. (2022). Fusing Bone-Conduction and Air-Conduction Sensors for Complex-Domain Speech Enhancement. IEEE/ACM Trans. Audio Speech Lang. Process..

[25] Luo Y., Mesgarani N. (2019). Conv-TasNet: Surpassing ideal time–frequency magnitude masking for speech separation. IEEE/ACM Trans. Audio Speech Lang. Process..

[26] Huang G., Liu Z., Maaten L.V.D., Weinberger K.Q. Densely Connected Convolutional Networks. Proceedings of the 2017 IEEE Conference on Computer Vision and Pattern Recognition (CVPR).

[27] Chen J., Mao Q., Liu D. (2020). Dual-Path Transformer Network: Direct Context-Aware Modeling for End-to-End Monaural Speech Separation. arXiv.

[28] Dai Y., Gieseke F., Oehmcke S., Wu Y., Barnard K. Attentional Feature Fusion. Proceedings of the 2021 IEEE Winter Conference on Applications of Computer Vision (WACV).

[29] Li A., Liu W., Zheng C., Fan C., Li X. (2021). Two heads are better than one: A two-stage complex spectral mapping approach for monaural speech enhancement. IEEE/ACM Trans. Audio Speech Lang. Process..

[30] Shi W., Caballero J., Huszar F., Totz J., Aitken A.P., Bishop R., Rueckert D., Wang Z. Real-Time Single Image and Video Super-Resolution Using an Efficient Sub-Pixel Convolutional Neural Network. Proceedings of the 2016 IEEE Conference on Computer Vision and Pattern Recognition (CVPR).

[31] Pandey A., Wang D. Densely Connected Neural Network with Dilated Convolutions for Real-Time Speech Enhancement in The Time Domain. Proceedings of the ICASSP 2020—2020 IEEE International Conference on Acoustics, Speech and Signal Processing (ICASSP).

[32] Valentini-Botinhao C., Wang X., Takaki S., Yamagishi J. Speech Enhancement for a Noise-Robust Text-to-Speech Synthesis System using Deep Recurrent Neural Networks. Proceedings of the INTERSPEECH 2016.

[33] Rix A.W., Beerends J.G., Hollier M.P., Hekstra A.P. Perceptual evaluation of speech quality (PESQ)-a new method for speech quality assessment of telephone networks and codecs. Proceedings of the 2001 IEEE International Conference on Acoustics, Speech and Signal Processing (ICASSP).

[34] Hu Y., Loizou P.C. (2008). Evaluation of objective quality measures for speech enhancement. IEEE/ACM Trans. Audio Speech Lang. Process..

